# The social determinants of mental health disorders among women with infertility: a systematic review

**DOI:** 10.1186/s12905-023-02828-9

**Published:** 2023-12-13

**Authors:** Tanmay Bagade, Amanual Getnet Mersha, Tazeen Majeed

**Affiliations:** School of Medicine and Public Health, College of Health, Medicine, and Wellbeing, The University of Newcastle, Callaghan, NSW 2285 Australia

**Keywords:** Infertility, Fertility, Reproductive health, Mental health, Depression, Anxiety, Women, Social determinants of health, Social support, SDoH, Socioeconomic, Socioeconomic

## Abstract

**Introduction:**

Infertility is associated with mental health disorders in women, even if a successful pregnancy resolves infertility. However, the link between social determinants of health (SDoH) and mental health in women with infertility is not well understood. We aimed to investigate the determinants thoroughly so that mental health screening and services can be tailored to suit women with infertility who are vulnerable to mental health disorders.

**Methodology:**

All observational studies that included women participants of reproductive age with infertility and assessed social determinants associated with mental health disorders were searched using a combination of keywords from MEDLINE, EMBASE, CINAHL, PsycINFO, Scopus, and Web of Science databases and published in English. Two reviewers conducted screening, data extraction, quality assessment and risk of bias. The protocol was registered on PROSPERO (number CRD42022343962).

**Results:**

The systematic review included 32 studies out of 3405 screened articles from January 1st 2010 to 16th October 2023. Compared to women without infertility, the prevalence of mental health disorders, including anxiety, depression, psychological distress, and stress, is high among women with infertility, with the severity being influenced by social determinants—those with higher education, employment, higher personal or family income, private health insurance, higher social support, stronger religious beliefs, and higher spiritual well-being reported better mental health outcomes.

**Conclusion:**

The study highlights the need for early detection, tailored interventions, and integrated and comprehensive support systems to address the mental health needs of women with infertility and improve their well-being.

**Supplementary Information:**

The online version contains supplementary material available at 10.1186/s12905-023-02828-9.

## Introduction

Infertility, the failure to achieve a pregnancy after one year of regular unprotected sexual intercourse, is an emerging global health issue [1–3]. Infertility can be primary (never achieved pregnancy) or secondary (woman achieves at least one pregnancy) [2, 3]. A comprehensive assessment of its global prevalence rate indicated that infertility has increased by 14.9% in 2017 compared to the global rates in 1990 and affects more than 10% of the world’s population [4, 5]. The 2018 Guttmacher-Lancet Commission report states that infertility affects between 49 and 180 million couples worldwide, and many lack access or availability to sexual and reproductive health services [6].

Sexual and reproductive health rights are essential for the progress of Sustainable Development Goals due to their association with gender equity, health, and well-being. Article 16 of the Human Rights Declaration states that “Men and women of full age, without any limitation due to race, nationality or religion, have the right to marry and to found a family” [7]. Therefore, infertility should be considered a human rights issue, and factors such as availability and access to infertility treatment, including in-vitro fertilisation, should be recognised as a human right [8].

Furthermore, although infertility can affect men and women, its sociocultural impact disproportionately affects women more than men because motherhood is perceived as an essential component of women’s identity, especially in low and middle-income countries [9, 10]. Having children is considered a crucial part of life for many people, and several societies value parenthood as a necessary achievement in marital relationships [11]. The centrality of motherhood in a sociocultural context is deep-rooted and may cause significant stress to a woman if she has not attained motherhood as age advances [9, 12]. Psychological stress can affect women’s physical and mental health, career choices, and overall well-being [14]. [13]

Infertility is unlike other conditions where a precise diagnosis can achieve a straightforward treatment. Infertility treatment such as in-vitro fertilisation (IVF) is costly, lengthy, and has unpredictable success rates in achieving a full-term and healthy pregnancy [15, 16]. The unreliability of treatment and financial cost associated with IVF can further escalate psychological stress. Failure of infertility treatment has also been shown to be emotionally distressing to women [17]. Furthermore, the relationship between infertility and psychological stress is bidirectional because infertility can lead to stress, and stress is also a risk factor for infertility [18]. Kiani et al. have estimated a 44.32% prevalence of depression and 54.24% prevalence of anxiety in women with infertility in low and middle-income countries and 28.03% prevalence for depression and 25.05% for anxiety in high-income countries [19, 20]. For instance, a recent longitudinal study by Bagade et al. reported a significant association between infertility and psychological distress in women, even after the infertility is resolved by a successful pregnancy, highlighting the long-term consequences of infertility on mental health [1]. Moreover, the mental health impact of infertility can vary based on individuals’ social support, socio-economic status, and coping mechanisms [21]. Collectively or independently, these social determinants of health (SDoH) can influence health outcomes [22–24]. Furthermore, SDoH also determines healthcare utilisation and adherence to treatment[25]. The World Health Organization defines social determinants of health as “*non-medical factors that influence health outcomes. They are the conditions in which people are born, grow, work, live, and age, and the wider set of forces and systems shaping the conditions of daily life. These forces and systems include economic policies and systems, development agendas, social norms, social policies and political systems*” [26]. This broad definition can include numerous non-clinical factors of health such as age, gender, ethnicity, education, income and social security, social support, food security, housing, job security, harmful alcohol consumption or tobacco addiction, etc. [26, 27]. However, a limited number of these SDoH such as education or income are regularly reported or studied in the literature [24, 28]. Furthermore, difficulty in obtaining information on reported SDoH from multiple sectors is a challenge [28]. In this systematic review, therefore, we aim to conduct an in-depth analysis of existing literature to understand the SDoH that can influence the mental health impact of infertility in women.

## Materials and methods

A systematic review was conducted to evaluate the social determinants associated with various mental health disorders among women with infertility. This review was done in accordance with the Preferred Reporting Items for Systematic Reviews and Meta-Analyses (PRISMA) guidelines [29]. A prior review protocol was prepared and registered in PROSPERO with a registration number of CRD42022343962 and can be accessed at https://www.crd.york.ac.uk/prospero/display_record.php?ID=CRD42022343962.

### Eligibility criteria

PICOS elements were used to set criteria for the inclusion and exclusion of studies in this review:

### Participants/population

Studies conducted among reproductive-aged women with infertility and assessed social determinants associated with any mental health conditions were included. Observational studies where study samples included the general population, as well as those with specific subpopulations such as women undergoing any infertility treatment such as Assisted Reproductive Technology (ART) treatments, studies conducted among women with resolved infertility, studies conducted in low socioeconomic status, or specific ethnic group were also included. Studies with participants who were already diagnosed with mental health conditions such as depression were excluded from this review.

### Intervention(s), exposure(s)

The review aimed at understanding the determinants of mental health conditions in a real-world context using observational studies with social determinants as the exposure variable. Interventional studies that evaluated the effectiveness of interventions targeted at improving the mental health conditions of women with infertility were excluded.

### Comparator(s)/control

Depending on the research objectives, studies comparing mental health conditions among women with and without infertility were included. Studies with a randomised trials design that compared control and treatment groups were excluded.

### Outcomes

The primary outcome of this review was to identify the social determinants of mental health disorders among women with infertility. The following social determinants were evaluated: age, income, employment status, education level, housing status, marital status, living condition (nuclear family, joint family where intergenerational people live together), social support from partner, family or friends, medical insurance, harmful alcohol consumption, tobacco addiction, and societal factors such as stigma, religion, and spirituality. Social determinants have been assessed through diverse methods. For instance, education has been evaluated in terms of educational level attained or just formal schooling. Similarly, income has been presented by earnings or social class categorisation. We included all variations and reporting of social determinants for our asessments.

### Types of studies to be included

Observational studies published since January 1st, 2010, were deemed eligible for inclusion. No exclusions were applied based on study area/location, but the search was limited to studies published in English. The following types of articles: reviews, commentaries, expert opinions, case reports, case series, case control, position statements, clinical trials, and conference abstracts, animal studies were excluded.

### Search strategies

A systematic approach was employed to conduct a literature search in multiple databases, subject-specific journals, and grey literature sources. Literature search was conducted on 11th June 2022 and updated on 16th October 2023 to retrieve recently published articles from the following six databases: MEDLINE, EMBASE, CINAHL, PsycINFO, Scopus, and Web of Science. The following combination of keywords and phrases were combined using Boolean logic operators: AND, OR, and NOT and used to retrieve citations: (mental health, mental illness, mental disorder, psychiatric illness, depression, depressive disorder, depressive symptoms, major depressive disorder, anxiety disorders, anxiety, generalised anxiety disorder, psychological distress, psychological stress, psychological distress, mental stress, bipolar disorder, bipolar I, bipolar II, manic depression, bipolar affective disorder, bipolar depression, mood disorders, psychosis, schizophrenia, psychotic disorder, severe mental illness, serious mental illness, somatic symptom disorder) AND (infertility, infertile, fertility issues, infertility in women, infertility treatment, infertility in couples, childlessness, Sterility, Reproductive Sterility, Sub-Fertility, Female Infertility, Female Subfertility, Female Sterility, Fecundability, Fecundity, Subfecundity). A complimentary search, including forward and backward citation searches of included articles, and free Google searches were also conducted to locate additional eligible articles. To provide contemporary evidence, the investigation included articles published from January 1, 2010 [Supplementary material—1].

### Citation screening

Retrieved citations from multiple sources were exported into Endnote referencing software version 9 and exported to Covidence software for screening [30]. The title and abstract of citations were screened by two reviewers independently. A third reviewer adjudicated the decision when there were conflicts. The full-text screening was conducted by two reviewers independently. Disagreements during full-text screening were resolved through discussion and mutual understanding.

### Quality assessment

The Joanna Briggs Institute (JBI) Critical Appraisal tools for observational studies [31] was used to evaluate the methodological rigour of the included studies. Two reviewers independently assessed the methodological quality for various criteria such as appropriate sampling, dealing with confounding factors, outcome measures, and appropriate statistical analysis. Conflicts on the quality of included studies were resolved by discussion and mutual agreement between the reviewers. Studies were appraised for each criterion, and the assessor could select ‘Yes’, ‘No’, ‘Unclear’ or ‘Not applicable’. The assessment outcomes were considered in synthesising and interpreting the finding of the review.

### Data extraction and synthesis

A data extraction template prepared in Microsoft Excel was used to extract relevant information from included studies on (1) Information on publication (name of the first author, year of publication), (2) study design (including aims, design, sample size, data collection method); (3) participant characteristics; (4) study location; (5) study setting (community or health setting based); (6) participant characteristics (mean age, gender, ethnicity);  (6) type of fertility; (7) details reported socioeconomic determinants (education, income, social support etc.); (8) reported mental health issue and (9) results of the study. Two reviewers (TB and TM) extracted, coded, and summarised the findings and different themes from included articles using NVivo statistical software [Supplementary material – 2]. Similarities and differences in the factors associated with mental health disorders among women with infertility were identified and discussed.

## Results

### Search results

**Fig. 1:**
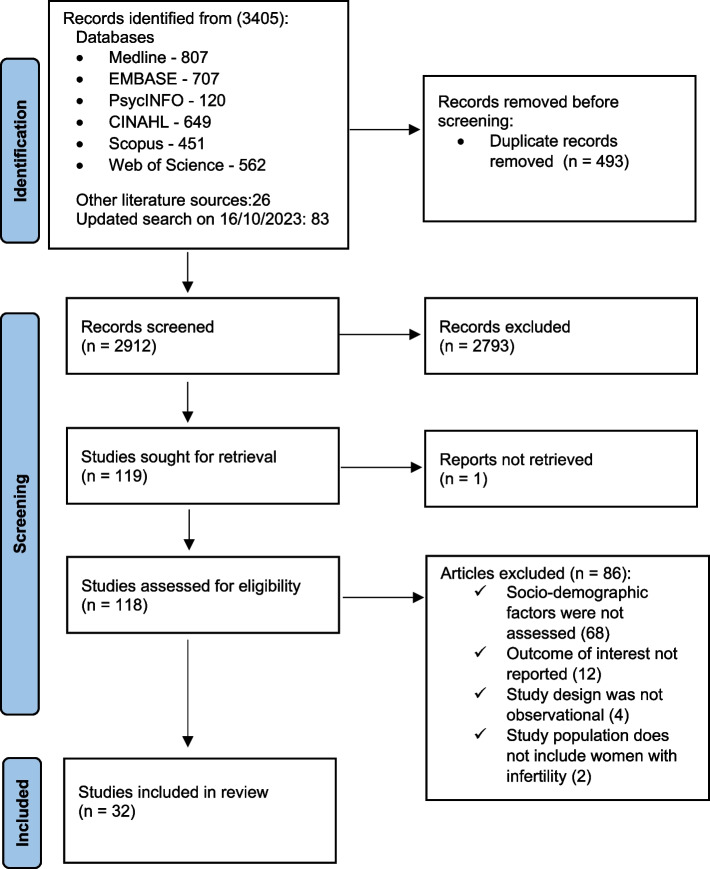
PRISMA Flow diagram of studies included in the review

An overview of the search results and the study selection process is outlined in Fig. 1 using the PRISMA flow diagram. The initial literature search yielded a total of 3405 citations. After excluding 493 duplicated citations, 2912 citations were screened using title and abstract for inclusion. A total of 118 citations were included in the full-text screening, and finally, 32 articles were deemed eligible for inclusion in the review after a rigorous and systematic screening process. The most common reason for exclusion was not reporting any social determinants of health.

### Characteristics of included studies

Most of the studies in the review reported a mixed sample of female participants with either ‘primary’ or ‘secondary’ infertility. Six studies included ‘only women’ with primary infertility [32–37]. Two studies included males and females [38, 39]. Studies conducted in Asia (*n* = 21, 63.6%) and Africa (*n* = 8, 24.2%) accounted for the majority of included studies. One study enrolled participants from the UK and Pakistan [40], while no studies were conducted in Australia and South America. All but one study used a cross-sectional study design to explore the social determinants associated with mental health disorders. One study used a retrospective cohort [38]. Nearly a third of the studies (*n* = 10, 31.3%) were conducted between 2010 and 2014; nine studies (28.1%) were conducted between 2015 and 2019; and the remaining (*n* = 13, 40.6%) were conducted between 2020 to 2022. Various instruments were used to evaluate participants’ mental health status, (*n* = 6, 18.7%) Beck Depression Inventory and the Depression Anxiety Stress Scale (*n* = 4, 12.5%) being the most utilised instruments (Table 1).

**Table 1:** Characteristics of the studies (*n* = 32) included in the review

Categories	Frequency (%)
**Type of participants considered in the studies:**	
Mixed primary and secondary infertility	26(81.3%)
Only Primary infertility	6(18.7%)
**Sex of participants:**	
Only women participants	30(93.8%)
Both men and women participants	2(6.2%)
**Publication years**	
2010—2014	10(31.3%)
2015 – 2019	9(28.1%)
2020 – 2023	13(40.6%)
**Reported mean age (Only studies that reported mean age)**	
< 30 years	7(33.3%)
≥ 30 years	14(66.7%)
**Study designs**	
Cross-sectional	31(96.9%)
Cohort/longitudinal	1(3.1%)
Location (region) of the studies^a^	
Asia	21(63.6%)
Africa	8(24.2%)
North America	2(6.1%)
South America	0(0%)
Europe	2(6.1%)
Australia	0(0%)
**Instruments used to measure mental health status by the studies**^**b**^	
Beck Depression Inventory (BDI)	6(18.7%)
Depression Anxiety Stress Scale (DASS)	4(12.5%)
Patient Health Questionnaire-9 (PHQ-9 scale)	3(9.3%)
Fertility-Specific Distress Scale	3(9.3%)
State-Trait Anxiety Inventory	2(6.2%)
Kessler Six-question Psychological Distress Scale (K-6 score)	2(6.2%)
General Health Questionnaire	2(6.2%)
The Copenhagen Multi‐Centre Psychosocial Infertility Stress questionnaire	2(6.2%)
Hospital Anxiety and Depression Scale (HADS)	2(6.2%)
General Health Questionnaire (GHQ)	2(6.2%)
Self-rating Depression Scale (SDS)	2(6.2%)
Hamilton Depression Rating Scale	1(3.1%)
Hamilton Anxiety Rating Scale	1(3.1%)
Post-traumatic growth (PTG) scale (21-item scale)	1(3.1%)
Zung’s self-rating depression assessment scale	1(3.1%)

^a^One study was conducted in the UK and Pakistan

^b^Some studies used more than one instrument to evaluate multiple mental health conditions

Generally, the methodological qualities of the included studies were assessed to have good quality by both reviewers. More detailed assessments of study qualities are illustrated in Supplementary Material 3. Most studies (*n* = 19) meet the JBI Critical Appraisal Checklists for cross-sectional studies. Eleven studies meet five to six criteria out of eight criteria. Two studies meet only two to four of the JBI quality assessment tool. The most common limitations identified by the reviewers were failure to identify or have a clear strategy to deal with confounding factors and not reporting details of study settings [Supplementary material – 3].

### Mental health outcomes assessed across studies

Among the women who reported infertility, 24% to 41.1% reported coexisting anxiety and depression [41, 42].

### Anxiety

Four studies [39, 41–43] assessed anxiety in individuals with infertility. Three of these studies included women with infertility [41–43], while study enrolled both men and women with infertility [39]. The prevalence of anxiety disorders associated with infertility ranged from 21.8% [44] to 27.5% [41]. Notably, primary infertility had higher anxiety rates (23.5%) compared to secondary infertility (18.4%) [44].

### Depression

Eleven studies reported the prevalence of depression among women with infertility [36, 41, 42, 45–52] with one focusing only on primary infertility [36]. The rates of mild to moderate depression ranged from 12.2% [51] to 80% [36] and severe depression between 4.27% [45] and 5.4% [46].

### Psychological distress

Four studies [32, 39, 52–54], including one that enrolled both men and women [39] used different assessment tools and reported psychological distress prevalence of 36.5% [54] 40.7% [39]. Psychological distress was also higher among women with infertility (66.1%) compared to those without infertility (18.6%) [32].

### Stress

Two studies [55, 56] evaluated stress in women with infertility using common mental health scales. They found high prevalence rates, with 92.7% experiencing infertility-related stress [55] and 50% having severe degree of stress [56].

### Social determinants associated with mental health disorders

#### Education

Lower levels of formal education were associated with higher rates of anxiety and depression [57]. The husband's lower educational level (less than 10 years) was associated with lower psychological distress in women.

#### Employment

Employment status had significant associations with various mental health conditions [47, 48, 53, 58–60]. Employed women had lower anxiety, depression and stress levels [47, 60]. Conversely, perceived difficulties to continue working during fertility treatment and infertility-related harassment in the workplace were associated with higher rates of psychological distress [53].

#### Income

Six studies assessed the association between income, salary levels, social class and mental health issues among women reporting infertility and reported significant associations [38, 43, 44, 50, 54, 55]. Lower income was associated with higher anxiety [44] and women from lower social class also scored high on the state anxiety scale (STAI) compared to women of medium and higher social classes [43]. Higher individual and family incomes reduced depression risk [45, 50], while inadequate family income was associated with higher rates of psychological distress [53]. Moreover, women who reported depression and anxiety indicated they faced significant financial problems due to infertility treatment [42].

#### Social support

Lack of social support was linked to anxiety, while support from family and friends reduced anxiety in women with infertility [41]. Social support was also a predictor of depression [41, 48, 61–63], and depression was associated with impaired social functioning [49]. Family dysfunction and poor husband support worsened depression severity [32, 46]. Social isolation was linked to depressive symptoms [52]. Adequate partner support and family support reduced distress [21, 35, 42, 55, 59]. Although family and friends' support is associated with a lower risk of psychological distress [35], family encouragement to seek treatment is associated with higher rates of distress [59]. Women with secondary infertility with live children were less likely to develop stress than women diagnosed with primary infertility [55, 64].

### Other factors associated with mental health

#### Stigma

Social stigma was associated with higher rates of anxiety and depression [41]. Social stigma was associated with higher rates of anxiety and depression [41]. Experiencing stigmatising behaviours (such as humiliation, discrimination, and devaluation) was associated with threefold higher rate of depression [41]. Chinese women with infertility reported discrimination, shame, and reproductive pressures associated with depression [45].

### Age

Increasing age correlated with higher depression scores [47, 65]. Younger women had less psychological distress [35], and older women (35 years and more) had more infertility-related stress [55].

### Religion and spirituality

Strong religious beliefs were associated with lower depression rates [60]. Religion also affected depression severity, with Muslims having higher severity than Christians [50]. Mental health problems were lower in women with higher religiosity [59]. Spiritual well-being was positively related to mental health [66].

### Alcohol

Depression in women with infertility was associated with an alcohol-addicted husband [51].

### Health insurance

Women with private health insurance had lower psychological distress risks [59].

### Living condition

Living in a joint family (multigenerational family living together) was a significant risk factor for anxiety and depression [42].

## Discussion

This systematic review investigated the associations of social determinants of health that affect the mental health outcomes of women with infertility. We found that women’s higher education, employment, higher personal or family income, private health insurance, higher social, partner, friends and family support, stronger religious beliefs and increased spiritual well-being were associated with better mental health outcomes in women with infertility. On the contrary, higher education of partners, social stigma, older age, and alcohol-addicted partner or husband were risk factors for developing mental health conditions in women with infertility. Social determinants of health play a vital role in determining an individual's health outcomes. To our knowledge, this is the first study to study the role of social determinants on the mental health outcomes of women with infertility.

The studies included in this systematic review reported a prevalence of 21.8% to 27.5% of anxiety and a 12.2% to 80% prevalence of depression in women with infertility [36, 41, 44, 51]. Previous meta-analysis has reported a similar 25.05% to 54.24% prevalence of anxiety and 28.03% to 44.32% prevalence of depression in women with infertility [19, 20].

It was not surprising to find that education was inversely associated with anxiety and depression in women with infertility. Education empowers women, makes them capable of interpreting information and coping with life’s stressors and is also associated with improvement in mental health service utilisation [67, 68]. However, we also found that a higher education level of partners was associated with higher psychological distress in women. Furthermore, the alcohol addiction of the partner was also associated with depression in women with infertility. These are concerning findings and require further evaluation to understand the influence of male partner’s social determinants and associated factors with women’s mental health.

Our review indicated that socioeconomic disparities significantly impact the mental health of women with infertility. Social inequality also affects the access and use of treatment services such as ART [16, 69]. Women with lower personal or family income and unemployment were found to have the highest prevalence of anxiety and depression. Financial constraints are a significant issue in accessing infertility treatment. Infertility treatment that includes various types of ART is expensive for couples, especially in countries where infertility treatment is not subsidised [15, 16]. In a cost-effectiveness study conducted in Australia, the average healthcare cost for ART live-birth events ranged from approximately 32,900 US dollars for women under 32 years to 182,000 US dollars for women aged 42 and older [15]. Employment improves women's income and financial autonomy and is associated with healthcare utilisation, especially for ART treatment [53, 70]. Previous studies have also implied that higher educational level, employment, and income are associated with a higher probability of accessing ART treatment [69].

Social isolation and stigma significantly affect one’s ability to cope with adversities and can cause adverse mental health impacts on women with infertility. Two studies from our review reported that social stigmas such as harassment, devaluation, and discrimination against women with infertility were significantly associated with depression [41, 45]. In another study, women with infertility living in joint families were subjected to higher stigmatisation and reported adverse mental health outcomes [42]. Conversely, social support, especially from partners and family, improves coping and response for women with infertility. Numerous other studies have confirmed that social support is vital for people to manage their chronic conditions, stress, and mental health conditions, such as anxiety and depression and improve their quality of life [71, 72].

Religiosity and spiritual health were associated with a lower prevalence of mental health disorders in women with infertility. Our review indicated that spiritual well-being is a significant factor in improving mental health. Inter-religious comparison showed Muslim women with infertility had higher rates of depression compared to non-Muslim women [50, 73]. Higher representation of Muslim women in the sample [50, 73] and cultural pressure of childbearing can be crucial factors of this association[50]. Spirituality and religiosity are associated with improved coping mechanisms, and can have a protective effect against suicide attempts and ideation, and better mental health outcomes longitudinally [74–76]. Furthermore, researchers have also highlighted that religious attendance and frequent engagement in prayers, meditation or religious activities facilitate improving mental health outcomes and quality of life [77–79]. Armah et al., in a systematic review, have highlighted the need to include spiritual interventions as an essential component of a holistic approach to managing female infertility [80]. However, few researchers have highlighted the challenges and heterogenicity in the studies assessing religiosity and spirituality on mental health outcomes. These studies reported a mixed or negative association between religiosity and mental health outcomes due to measurement issues and a wide variation in the religious or spiritual beliefs and degrees of secularity, such as cultural and societal norms [81].

This systematic review has a unique approach with several strengths. We have used a robust methodology of systematically analysing the literature. Due to the inclusion of social determinants of health framework, the study adds a critical and unique perspective to the existing body of evidence about mental health issues in women with infertility. The study is not without limitations, mainly due to the inconsistency and heterogenicity of the social determinants reported by the articles, which makes it difficult to conduct a meta-analysis. A primary and significant limitation of the studies included in this review is that they are observational. Consequently, a true cause-and-effect relationship cannot be established. However, the robust systemic approach and focus on including only peer-reviewed journal articles in the review have minimised these limitations. Secondly, our analysis is based on multiple international sites with very different sociocultural contexts. However, not all SDOHs are going to translate across cultures (e.g., views of women’s education/empowerment, stigma associated with mental health, and the roles of issues such as medical racism), and we acknowledge that this is a limitation of reviewing a series of studies from such broad international samples. Furthermore, this review exclusively encompassed articles published in the English language, primarily due to budgetary limitations, potentially overlooking pertinent research published in other languages.

The focus of infertility management should have a holistic and human rights approach complementing the medical treatment [80, 82, 83]. Researchers have advocated for increased government support to facilitate access subsidised treatment of infertility and reduce out-of-pocket expenses and financial burden borne by individual [82–84]. Government policies offering such support can significantly alleviate the financial stress associated with infertility treatments and, in turn, contribute to the reduction of psychological distress in women.

Furthermore, the persistent stigma surrounding women with infertility remains a global concern, linked to deeply ingrained societal, cultural, and religious influences [5, 71, 80, 85]. Enhancing mental health support for these women, with a strategic emphasis on cultivating effective coping mechanisms, is essential for empowering them to navigate the psychological stress associated with infertility, benefiting both themselves and their families [21, 60, 85]. The social support from families, friends and health professionals might be inadequate, therefore, researchers have recommended establishing peer support groups [86]. Further studies should be conducted to develop an in-depth understanding of the coping strategies in different religions and how they can positively impact the mental health of women with infertility issues. Research should also place a strong emphasis on finding cost-effective and readily available infertility treatments.

In conclusion, infertility needs holistic intervention approaches that should extend beyond clinical care by considering the social determinants of health. Government and non-government services should work cohesively to identify women who are at increased risk of stress associated with infertility and provide an integrated support.

### Supplementary Information

**Additional file 1: Supplementary material 1. **Search strategies.

## Data Availability

The supplementary material contains all data associated with the manuscript. A prior review protocol was prepared and registered in PROSPERO with a registration number of CRD42022343962 and can be accessed at https://www.crd.york.ac.uk/prospero/display_record.php?ID=CRD42022343962.
