# Supplementary material for: The social determinants of mental health disorders among women with infertility: a systematic review
Source: BMC Womens Health. 2023 Dec 13;23:668. doi: 10.1186/s12905-023-02828-9 (PMC10720205; doi:10.1186/s12905-023-02828-9)
Supplement: Supplementary file 1 — Additional file 1: Supplementary material 1. Search strategies. [file 12905_2023_2828_MOESM1_ESM.docx]

**Supplementary Material**

**Supplementary material – 1 - Search strategies**

**1. MEDLINE**

| 1. | affective symptoms/ or depression/ or mental fatigue/ or obsessive behavior/ or paranoid behavior/ or schizophrenic language/ or stress, psychological/ |
| --- | --- |
| 2. | depressive disorder/ or depressive disorder, major/ or personality disorders/ or "schizophrenia spectrum and other psychotic disorders"/ or somatoform disorders/ |
| 3. | anxiety disorders/ or anxiety, separation/ or obsessive-compulsive disorder/ or panic disorder/ or "bipolar and related disorders"/ or "disruptive, impulse control, and conduct disorders"/ or dissociative disorders/ or elimination disorders/ or "feeding and eating disorders"/ or mood disorders/ or depressive disorder/ or cyclothymic disorder/ or motor disorders/ or personality disorders/ or "schizophrenia spectrum and other psychotic disorders"/ or affective disorders, psychotic/ or paranoid disorders/ or psychotic disorders/ or schizophrenia/ |
| 4. | mental disorders/ or anxiety disorders/ or "bipolar and related disorders"/ or "disruptive, impulse control, and conduct disorders"/ or dissociative disorders/ or elimination disorders/ or "feeding and eating disorders"/ or mood disorders/ or personality disorders/ or "schizophrenia spectrum and other psychotic disorders"/ or somatoform disorders/ or substance-related disorders/ or "trauma and stressor related disorders"/ |
| 5. | mood disorders/ or motor disorders/ |
| 6. | Somatoform Disorders/di [Diagnosis] |
| 7. | stress, psychological/ or burnout, psychological/ or financial stress/ |
| 8. | affective disorders, psychotic/ or paranoid disorders/ or psychotic disorders/ or psychoses, substance-induced/ or schizophrenia/ |
| 9. | "bipolar and related disorders"/ or bipolar disorder/ |
| 10. | infertility/ or infertility, female/ |
| 11. | Fecundability.mp. |
| 12. | Reproductive Sterility.mp. |
| 13. | Childlessness.mp. |
| 14. | 1 or 2 or 3 or 4 or 5 or 6 or 7 or 8 or 9 |
| 15. | 10 or 11 or 12 or 13 |
| 16. | 14 and 15 |
| 17. | limit 16 to (english language and humans and yr="2010 -Current") |

**2. PSYCHINFO**

| 1. | affective symptoms/ or depression/ or mental fatigue/ or obsessive behavior/ or paranoid behavior/ or schizophrenic language/ or stress, psychological/ |
| --- | --- |
| 2. | depressive disorder/ or depressive disorder, major/ or personality disorders/ or "schizophrenia spectrum and other psychotic disorders"/ or somatoform disorders/ |
| 3. | anxiety disorders/ or anxiety, separation/ or obsessive-compulsive disorder/ or panic disorder/ or "bipolar and related disorders"/ or "disruptive, impulse control, and conduct disorders"/ or dissociative disorders/ or elimination disorders/ or "feeding and eating disorders"/ or mood disorders/ or depressive disorder/ or cyclothymic disorder/ or motor disorders/ or personality disorders/ or "schizophrenia spectrum and other psychotic disorders"/ or affective disorders, psychotic/ or paranoid disorders/ or psychotic disorders/ or schizophrenia/ |
| 4. | mental disorders/ or anxiety disorders/ or "bipolar and related disorders"/ or "disruptive, impulse control, and conduct disorders"/ or dissociative disorders/ or elimination disorders/ or "feeding and eating disorders"/ or mood disorders/ or personality disorders/ or "schizophrenia spectrum and other psychotic disorders"/ or somatoform disorders/ or substance-related disorders/ or "trauma and stressor related disorders"/ |
| 5. | mood disorders/ or motor disorders/ |
| 6. | Somatoform Disorders/di [Diagnosis] |
| 7. | stress, psychological/ or burnout, psychological/ or financial stress/ |
| 8. | affective disorders, psychotic/ or paranoid disorders/ or psychotic disorders/ or psychoses, substance-induced/ or schizophrenia/ |
| 9. | "bipolar and related disorders"/ or bipolar disorder/ |
| 10. | infertility/ or infertility, female/ |
| 11. | Fecundability.mp. |
| 12. | Reproductive Sterility.mp. |
| 13. | Childlessness.mp. |
| 14. | 1 or 2 or 3 or 4 or 5 or 6 or 7 or 8 or 9 |
| 15. | 10 or 11 or 12 or 13 |
| 16. | 14 and 15 |
| 17. | limit 16 to (english language and humans and yr="2010 -Current") |

**3. EMBASE**

| 1. | affective symptoms/ or depression/ or mental fatigue/ or obsessive behavior/ or paranoid behavior/ or schizophrenic language/ or stress, psychological/ |
| --- | --- |
| 2. | depressive disorder/ or depressive disorder, major/ or personality disorders/ or "schizophrenia spectrum and other psychotic disorders"/ or somatoform disorders/ |
| 3. | anxiety disorders/ or anxiety, separation/ or obsessive-compulsive disorder/ or panic disorder/ or "bipolar and related disorders"/ or "disruptive, impulse control, and conduct disorders"/ or dissociative disorders/ or elimination disorders/ or "feeding and eating disorders"/ or mood disorders/ or depressive disorder/ or cyclothymic disorder/ or motor disorders/ or personality disorders/ or "schizophrenia spectrum and other psychotic disorders"/ or affective disorders, psychotic/ or paranoid disorders/ or psychotic disorders/ or schizophrenia/ |
| 4. | mental disorders/ or anxiety disorders/ or "bipolar and related disorders"/ or "disruptive, impulse control, and conduct disorders"/ or dissociative disorders/ or elimination disorders/ or "feeding and eating disorders"/ or mood disorders/ or personality disorders/ or "schizophrenia spectrum and other psychotic disorders"/ or somatoform disorders/ or substance-related disorders/ or "trauma and stressor related disorders"/ |
| 5. | mood disorders/ or motor disorders/ |
| 6. | Somatoform Disorders/di [Diagnosis] |
| 7. | stress, psychological/ or burnout, psychological/ or financial stress/ |
| 8. | affective disorders, psychotic/ or paranoid disorders/ or psychotic disorders/ or psychoses, substance-induced/ or schizophrenia/ |
| 9. | "bipolar and related disorders"/ or bipolar disorder/ |
| 10. | infertility/ or infertility, female/ |
| 11. | Fecundability.mp. |
| 12. | Reproductive Sterility.mp. |
| 13. | Childlessness.mp. |
| 14. | 1 or 2 or 3 or 4 or 5 or 6 or 7 or 8 or 9 |
| 15. | 10 or 11 or 12 or 13 |
| 16. | 14 and 15 |
| 17. | limit 16 to (human and english language and embase and yr="2010 -Current" and article) |

**4. CINAHL Complete**

1. ( mental health or mental illness or mental disorder or psychiatric illness ) OR ( depression or depressive disorder or depressive symptoms or major depressive disorder ) OR ( anxiety disorders or anxiety or generalized anxiety disorder ) OR psychological distress OR ( psychological stress or psychological distress or mental stress or mental health ) OR ( bipolar disorder or bipolar i or bipolar ii or manic depression or bipolar affective disorder or bipolar depression ) OR mood disorders OR ( psychosis or schizophrenia or psychotic disorder ) OR ( psychosis or schizophrenia or psychotic disorder or severe mental illness or serious mental illness or mental illness ) OR somatic symptom disorder

**AND**

1. (infertility or infertile or fertility issues ) OR infertility in women OR infertility treatment OR infertility in couples OR ( infertility or fertility treatment or reproductive ) OR infertility psychological impact OR ( infertility and mental health )

**5. Web of Science**

1. ALL=(1. ( mental health or mental illness or mental disorder or psychiatric illness ) OR ( depression or depressive disorder or depressive symptoms or major depressive disorder ) OR ( anxiety disorders or anxiety or generalized anxiety disorder ) OR ( psychological stress or psychological distress or mental stress or mental health ) OR ( bipolar disorder or bipolar i or bipolar ii or manic depression or bipolar affective disorder or bipolar depression ) OR mood disorders OR ( psychosis or schizophrenia or psychotic disorder ) )

AND

1. ALL=(2. (infertility or infertile or fertility issues ) OR infertility in women OR infertility treatment OR infertility in couples OR ( infertility or fertility treatment or reproductive ) OR infertility psychological impact OR ( infertility and mental health ))

**6. Scopus**

( ALL ( ( mental  AND health  OR  mental  AND illness  OR  mental  AND disorder  OR  psychiatric  AND illness )  OR  ( depression  OR  depressive  AND disorder  OR  depressive  AND symptoms  OR  major  AND depressive  AND disorder )  OR  ( anxiety  AND disorders  OR  anxiety  OR  generalized  AND anxiety  AND disorder )  OR  ( psychological  AND stress  OR  psychological  AND distress  OR  mental  AND stress  OR  mental  AND health )  OR  ( bipolar  AND disorder  OR  bipolar  AND i  OR  bipolar  AND ii  OR  manic  AND depression  OR  bipolar  AND affective  AND disorder  OR  bipolar  AND depression )  OR  mood  AND disorders  OR  ( psychosis  OR  schizophrenia  OR  psychotic  AND disorder ) ) )

AND  ( ALL ( ( infertility  OR  infertile  OR  fertility  AND issues )  OR  infertility  AND in  AND women  OR  infertility  AND treatment  OR  infertility  AND in  AND couples  OR  ( infertility  OR  fertility  AND treatment  OR  reproductive )  OR  infertility  AND psychological  AND impact  OR  ( infertility  AND  mental  AND health ) ) )

**Supplementary material – 2 - Summary of included studies**

| **Source** | **Study aim** | **Location** | **Setting** | **Study design** | **Participant characteristics** | **Type of infertility** | **Sample size** | **Instrument used** |
| --- | --- | --- | --- | --- | --- | --- | --- | --- |
| Adelosoyo 2020 (Adelosoye et al. 2020) | To assess the impact of family function and husband support on depression severity among women with infertility. | Nigeria | Teaching hospital | cross-sectional | Female with primary and secondary infertility, mean age: 36 ± 5.3 years age, tertiary education, married, employed, family functioning, husband support. | Primary and secondary infertility | 341 | Zung’s self-rating depression assessment scale |
| Alhassan 2014 (Alhassan et al. 2014) | To examine prevalence and severity of depression in relation to age, type of infertility and duration of infertility in women with infertility. | Ghana | Teaching hospital | cross-sectional | The mean age: 30.5 ± 6.3 years age. Majority of the women 48.0% (48/100) were within 20 to 30 years age group followed by those in the 31-to-35-year age group (32.0%). (14.0%) were unemployed as shown. Over 80.0% of the women practice the Islamic faith with. The majority (60.0%) were low-income earners. 54.0% of the study women had not attained any form of formal education with only 46% attaining at least basic formal education. | Primary and secondary infertility | 100 | Beck Depression Inventory |
| Batool 2014 (Batool and de Visser 2014) | To assess the impact of emotional intelligence, social support and contextual factors on the general health in women with infertility. | UK and Pakistan | Fertility clinic (UK) and hospitals (Pakistan | cross-sectional | Women with infertility in the age group between 21–45 years of age. Women with both primary and secondary infertility were included in the study. | Primary and secondary infertility | 312 : 148 (UK) 164 (Pakistan) | General Survey: Health Questionnaire GHQ-28, and The scale of emotional intelligence, Berlin Social Support Scale, women’s relationship satisfaction |
| Beygi 2021 (Beygi et al. 2021) | To investigate the relationship between spiritual wellbeing, mental health and quality  of life among women with infertility. | Iran | Teaching hospital | cross-sectional | Half of the participants were 30 to 39 years old. 37.7% were native to Shiraz, and the rest were non-native. In terms of education, 36.4% had diploma degrees, and 70% had a family of two members. | Primary and secondary infertility | 247 | Palutzian & Ellison 20-question (spiritual health), DASS-21 Questionnaire (mental health), FertiQoL questionnaire spiritual wellbeing questionnaire, |
| Dadhwal 2021 (Dadhwal et al.) | To assess the prevalence of depression  and anxiety in women with infertility and elucidate the psychosocial factors. | India | Tertiary hospital | cross-sectional | The mean age of the participants was 29.21 ± 3.74 years, and 121 (80.7%) women reported primary infertility. The average duration of infertility was approximately 5 years. Furthermore, 3.5 years was the average duration for which women had been seeking treatment. | Primary and secondary infertility | 150 | Hamilton Depression Rating Scale and Hamilton Anxiety Rating Scale, Rosenberg Self-Esteem  Scale, WHO-Quality of life-BREF, and Brief-COPE  Inventory. |
| Erdem 2014 (Erdem and Apay 2014) | To determine the relationship between perceived social support and depression among women with infertility. | Turkey | Fertility clinic | cross-sectional | The mean age of participants were 31.9 ± 6.2 years, employed 32%, marriage duration 8.2 years. Women with both primary and secondary infertility were included in the study. | Primary and secondary infertility | 238 | Beck Depression Inventory and the Multidimensional Scale of Perceived Social Support |
| Greil 2016 (Greil et al. 2016) | To explore whether fertility-specific distress varied by race/ethnicity among a nationally representative sample of US women. | USA | Data source: The National Survey of Fertility Barriers | cross-sectional | The sample for this study consisted of 2363 women, 53.6 percent White (n = 1266), 24.1 percent Black (n = 569), 19.2 percent Hispanic (n = 453), and 2.2 percent Asian (n = 51) women who ever experienced a period of at least 1 year of regular unprotected intercourse without conception (the medical definition of infertility) | Primary infertility | 2363 | Fertility-Specific Distress Scale |
| Greil 2011 (Greil et al. 2011) | To examine variation in fertility-specific distress (FSD) and general distress according to different experiences of infertility among US women. | USA | Data source: The National Survey of Fertility Barriers | cross-sectional | Age 25-45 years, prior pregnancy (secondary infertility, n 628) to women with infertility with no prior pregnancies (primary infertility, n 399). | Primary and secondary infertility | 4796 | General distress - CESD-10, fertility-specific distress |
| Gui 2021 (Gui et al. 2021) | To determine the prevalence of anxiety and its potential risk factors among Chinese women with infertility after the enforcement of ‘two-child policy’. | China | Reproductive Medical Centres | cross-sectional | The age of the study population ranged from 24 to 48 years old with a mean ± standard deviation of 35.26 ± 4.30 years.  Women with both primary and secondary infertility were included in the study. | Primary and secondary infertility | 693 | Generalized Anxiety Disorder-7 (GAD-7). |
| Honarvar 2020 (Honarvar and Taghavi 2020) | The goal of this study is to measure the influence of age, job status, and education level on both scores within Iranian women with infertility. | Iran | Government and private hospitals | cross-sectional | Age: mean age, 35.96; range, 26-45, education level: 42.5% Masters of Science, job status: 50.4% unemployed. | Primary and secondary infertility | 1000 | Beck Depression Index, and the Way of Religious Coping Scale (WORCS) |
| Hui 2017 (Li et al. 2017) | To explore the sociocultural factors influencing depression in Chinese women with infertility. | China | Hospitals | cross-sectional | Age of the participants ranged from 19 to 46, with an average of 29.15 ± 4.37 years. Duration of infertility ranged from 1209 months, with an average of 33.97 ± 38.97 months.  Studies included female patients diagnosed with infertility by specific causes (such as pelvic inflammatory disease, uterine myoma, endometriosis, or ovulation dysfunction), while their husbands were not diagnosed with infertility. | Primary and secondary infertility | 211 | Social Support Rating Scale (SSRS), and Self-rating Depression Scale (SDS) |
| Ikemoto 2021 (Ikemoto et al. 2021) | To identify risk factors for severe psychological stress in women undergoing fertility treatment. | Japan | Fertility clinics | cross-sectional | The participants’ age ranged from 26 to 45 years (Mean ± Std = 35.96 ± 4.46), with median and mode of 36. high school diploma, technician, Bachelor of Science (BSc) and Master of Science (MSc) and Philosophy Doctorate (PhD) is 21.6%, 21.6%, 42.8%, and 35%, respectively. unemployed and employed is 50.4% and 49.6%, respectively. | Primary and secondary infertility | 1672 | Kessler Six-question Psychological Distress Scale (K-6 score) |
| Khadim 2019 (Khadim et al. 2019) | To determine the relationship between perceived social support and mental health problems in women with primary and secondary infertility. | Pakistan | Fertility clinics | Cross-sectional | 150 infertile women (87 primary and 63 secondary) with the age range of 21 to 40 (Mean 28.89 SD 5.05). | Primary and secondary infertility | 150 | Multidimensional Scale of Perceived Social Support and Depression Anxiety Stress Scale |
| Khalid 2020 (Khalid and Dawood 2020) | To investigate the relationship of social support, self-efficacy, and cognitive coping with psychological distress, as well as, to determine the mediating role of self-efficacy and cognitive coping between social support and psychological distress. | Pakistan | Hospitals | cross-sectional | Age range of the participants was 21–40 years with an average of 29.08 years. The mean duration of marriage was 5.59 years (2–14 years) and the mean duration of treatment was 4.05 years (1–12 years). Mostly infertile women were undergraduate (55%); had no self-employment (74%) and had monthly income less than or equal to 20,000 “PKR” (39%). | Primary and secondary infertility | 158 | Multidimensional scale of perceived social support, infertility self-efficacy scale, coping strategies questionnaire and depression anxiety stress scale |
| Lanskara 2011 (Lansakara et al. 2011) | To investigate the psychological wellbeing and its correlates among Sri Lankan women with primary infertility. | Sri Lanka | District administrative unit | cross-sectional | Primary infertility, age 49.7% from 25-34 years old, 27.1% employed, 59.9% Buddhist, 28.2% experienced adverse life events, 2.3% history of mental illness | Primary infertility | 177 | The General Health Questionnaire-30 and Mental Health subcomponents of the Short Form-36 (SF-36) |
| Lykeridou 2011 (Lykeridou et al. 2011) | To examine the association between (1) occupational social class and coping responses, (2) coping responses and infertility-related stress and (3) occupational social class and infertility-related distress. | Greece | Public Clinics | cross-sectional | The mean age was 36.9 years. The mean duration of infertility was reported to be 5.17 (SD 3.9) years. Most women (95%) reported having no children and only 5% of women had at least one child. | Primary and secondary infertility | 404 | State-Trait Anxiety Inventory), infertility-related stress (Copenhagen Multi-centre Psychosocial Infertility) and coping strategies (Copenhagen Multi-centre Psychosocial Infertility |
| Naab 2013  (Naab et al. 2013) | To describe infertile women's psychosocial health problems and their infertility-related beliefs and examine the relationships between their beliefs about infertility and psychosocial health problems. | Ghana | Hospitals | cross-sectional | The majority of participants were natives of southern Ghana, between the ages of 30 and 39 years, and had high school or higher education. More than half (62%) of the women had a monthly income of 300 cedis ($200). The majority of the women (87%) were married, and 85% were in monogamous marriages. Ninety-one percent were Christians.  Three fourths (72%) of the women had secondary infertility, and 28% had primary infertility. The majority (76.6%) of the women reported receiving medical treatment, 8.9% reported using traditional treatment, and 1.6% reported using both. The duration of medical treatment was 1 year or less for 57%, 2 to 3 years for 27%, and 4 or more years for 16%. | Primary and secondary infertility | 203 | Fertility Belief Questionnaire |
| Namdar et al, 2017  (Namdar et al. 2017) | To examine health related QOL in women with infertility referring to infertility clinics in Shiraz, Iran. | Iran | Fertility clinics | cross-sectional | Women, 29.4 ± 5.4 years age, married for an average of 6.6 ± 0.5 years.101 (69.1%) were homemakers and 57 (39.0%) were employed. While 56 patients (38.3%) were from Shiraz, 67 (45.9%) were living in neighbouring towns, and 23 (15.8%) came from rural areas. | Primary infertility | 161 | Quality of Life Questionnaire for Infertile Couples, designed by Yaghmai et al. and General Health Questionnaire (GHQ). |
| Ogawa, 2011  (Ogawa et al. 2011) | To investigate factors leading to the anxiety, depression, and stress of patients receiving outpatient treatment, and compared the results by stratifying factors such as age and duration of infertility. | Japan | Hospital | Cross-sectional | Average age of participants were 34.5 ± 4.5 years and average duration of infertility was 3.3 ± 2.8 years. | Primary and secondary infertility | 83 | Self-rating Depression Scale (SDS) and the Hospital Anxiety and Depression Scale (HADS) test. |
| Oladeji, 2017  (Oladeji and OlaOlorun 2018) | To assess the prevalence and associated factors of depression among women with infertility. | Nigeria | Teaching Hospital | cross-sectional | Females, mean age of 34.5 ± 5.7 years. Women with complaints of failure to achieve conception after at least a year of adequate unprotected heterosexual coitus, whether or not they had had a child previously. | Primary and secondary infertility | 110 | Patient Health Questionnaire-9 |
| Omoaregba, 2011  (Omoaregba et al. 2011) | To determine the prevalence of psychological distress as well as its associated sociocultural characteristics among women attending the infertility clinic of a tertiary hospital in Nigeria. | Nigeria | Infertility clinic | cross-sectional | Females, Mean age fertility group 35.8 ± 5.9 years age and Mean age comparison group 30.6 ± 3.7 years age. | Primary and secondary infertility | 100 | General Health Questionnaire |
| Qadir, 2015  (Qadir et al. 2015) | To identify prevalence rates of psychological distress among Pakistani women seeking help for primary infertility. The associations of social support, marital adjustment, and sociodemographic factors with psychological distress were also examined. | Pakistan | Fertility clinics | cross-sectional | Females (20 - 41 years of age), 50% married for a maximum of 5 years. Women with primary infertility were included in the study. | Primary infertility | 177 | Multidimensional Scale of Perceived Social Support, and the Locke-Wallace Marital Adjustment Test |
| Razzaque, 2021  (Razzaque et al. 2021) | To assess the level of depression, anxiety, and stress among women with infertility and its relationship with the type of infertility and demographic attributes. | Pakistan | Hospital | cross-sectional | Females (20 - 45 years of age); 52.7% women were aged between 31 - 40 years. 75% were educated; 66.5% were housewives; 61% were married for 10 or more years with household income of >30 thousand PKR.  Primary infertility (n=100); Secondary infertility (n=100). | Primary and secondary infertility | 200 | The DASS (Depression, Anxiety Stress Scale) |
| Rufai, 2022  (Rufai et al.) | To assess the association between family functionality, sociodemographic factors, and depression severity in women with infertility attending a gynaecology clinic in northwest Nigeria. | Nigeria | Teaching hospital | cross-sectional | Women with primary or secondary infertility were included in the study. Females (15 - 49 years), Mean age=30.9 ± 6.6 years. | Primary and secondary infertility | 415 | Beck’s Depression Inventory and Family APGAR (Adaptability, Partnership, Growth, Affection, Resolve) questionnaires |
| Shafierizi, 2022 (Shafierizi et al.) | This study aimed to explore levels of post-traumatic growth (PTG) and anxiety/depressive symptoms and the role of personality traits, resilience, and social support as predictors of PTG in infertile men/women. | Iran | Infertility and Reproductive Health research Centre | cross-sectional | Male and female, aged between 18 to 45 years (MD = 29.72 ± 6.2). The mean age of men and women was 32.57 ± 5.06 and 28.78 ± 6.2, respectively. The majority of the women were housewives (77.9%). | Primary and secondary infertility | 162 (M=40; F=122) | Questionnaires: post-traumatic growth (PTG) scale (21 item scale), State-Trait Anxiety Inventory, Fertility Problem Inventory, NEO Five-Factor Inventory, Perceived Social Support, Kobasa hardiness short 20-item and Beck Inventory Depression. |
| Shin, 2021  (Shin et al. 2021) | To describe the quality of life (QOL) of Korean women experiencing infertility based on sociodemographic and infertility characteristics and to examine the associations among symptoms of depression, social support, and QOL. | Korea | Website | cross-sectional | Female (≥19 years); Primary infertility; 46.2% were aged 30 - 35 years, 44.3% were married for 2 to 4 years, 74.2% graduated from universities, 49.5% worked in offices, with remainder housewives. Most participants reported no known reason for infertility , and the most common time since infertility diagnosis was less than 1 year (36.6%), followed by 1 to 2 years (33.5%). | Primary infertility | 186 | Patient Health Questionnaire, Multidimensional Scale of Perceived Social Support, and the Fertility Quality of Life (FertiQOL) scale. |
| Sulyman, 2019 (Sulyman et al. 2019) | To determine the prevalence of anxiety and depressive disorders among women attending fertility clinic at Abubakar Tafawa Balewa University Teaching Hospital Bauchi, Nigeria. It also examined sociodemographic and clinical variables that are predictive of presence of these disorders. | Nigeria | Teaching hospital | cross-sectional | Female, between 19 and 43 years with the mean age of 29.6 years (SD ± 4).  The predominant type of infertility (60%) reported among the studied group was primary infertility. 80% of reported duration of infertility of < 10 years. The commonest cause of infertility was tubal/uterine factors. 58% were on treatment for ≤12 months and 8.7% on treatment for 5 or more years. 69.8% of the respondents with depression had primary infertility as against 57.1% among respondents without depression. | Primary and secondary infertility | 207 | HADS scale |
| Takaki, 2014  (Takaki and Hibino 2014) | To investigate how family-related opinions and stressful situations are related to psychological distress in women undergoing infertility treatment. | Japan | Fertility clinics | cross-sectional | Age 36.4 ± 4.5 years, marriage 5.5 ± 3.6 years, fertility treatment median 2 years (0-20 years). | Primary and secondary infertility | 635 | Kessler Six-question Psychological Distress Scale (K6) |
| Teklemicheal, 2022  (Teklemicheal et al. 2022) | To determine the magnitude of infertility related psychological stress and explore for associated factors among Ethiopian women with infertility seeking treatment. | Ethiopia | Hospitals | cross-sectional | Females, undergoing a non-IVF treatment for infertility. The mean age of participants was 31.5 (SD±5.9) years and the majority of women (58.3%) belonged to the 26–30 (33.3%) and 31–35 (25%) age categories. | Primary and secondary infertility | 96 | The Copenhagen Multi‐Centre Psychosocial Infertility-Fertility Problem Stress questionner |
| Tola, 2020  (Tola et al. 2020) | To investigate the relationship between infertility and Type D personality—defined as the presence of two personality characters, namely negative affectivity (NA) and social inhibition (SI) | Turkey | Fertility clinics | cross-sectional | Females, primary unexplained infertile comprising the infertile group and 156 age- and body mass index (BMI)-matched women diagnosed as fertile comprising the fertile group. The age of women with infertility ranged between 20 and 44 years (31.55 ± 5.24 years). The mean marriage duration of infertile group was 6.16 years (±4.42 years). | Primary infertility | 324 [168 infertile and 156 fertile controls] | The Turkish versions of the DS14 and the Beck Depression Inventory (BDI-21). |
| Vo, 2019  (Vo et al. 2019) | To determine the depression prevalence in women with infertility and its associated factors. | Vietnam | Fertility clinics | cross-sectional | Women with primary or secondary infertility were included in the study. The mean age of the participants was 30.41 years (SD=4.47). 87.8% were of the nulliparous group (primary infertility), while 12% belonged to the parous group (secondary infertility). | Primary and secondary infertility | 401 | The PHQ-9 scale. |
| Wang, 2018  (Wang et al. 2018) | To examine the factors for the development of mental disorders in women with infertility and measured the duration until the occurrence of mental disorders (outcome variable) after a diagnosis of infertility. | Taiwan | Data source: Taiwan National Health Insurance Research Database (NHIRD) | cohort study | Women and men with primary or secondary infertility were included in the study. The majority of participants were women (82.5%) aged 25–34 years (59.80%). | Primary and secondary infertility | 13,317 (Men - 17.4%, Female – 82.5%) | Not stated |

**Supplementary material 3:** **JBI Critical Appraisal Checklist for cross-sectional studies (Yes; No; Unclear (UC); Not applicable (NA))**

| 1. Were the criteria for inclusion in the sample clearly defined? 2. Were the study subjects and the setting described in detail? 3. Was the exposure measured in a valid and reliable way? 4. Were objective, standard criteria used for measurement of the condition? 5. Were confounding factors identified? 6. Were strategies to deal with confounding factors stated? 7. Were the outcomes measured in a valid and reliable way? 8. Was appropriate statistical analysis used? | | | | | | | | | |
| --- | --- | --- | --- | --- | --- | --- | --- | --- | --- |
| **Source** | **1** | **2** | **3** | **4** | **5** | **6** | **7** | **8** | Total |
| Adelosyo 2020 | Yes | Yes | Yes | Yes | Yes | Yes | Yes | Yes | 8 |
| Alhassan 2014 | Yes | Yes | Yes | Yes | Yes | Yes | Yes | Yes | 8 |
| Batool2014 | Yes | Yes | Yes | Yes | Yes | Yes | Yes | Yes | 8 |
| Beygi 2021 | Yes | Yes | Yes | Yes | Yes | Yes | Yes | Yes | 8 |
| Dadhwal 2021 | Yes | Yes | Yes | Yes | Yes | Yes | Yes | Yes | 8 |
| Erdem2014 (Erdem and Apay 2014) | Yes | Yes | Yes | Yes | Yes | Yes | Yes | Yes | 8 |
| Greil 2016 | Yes | Yes | Yes | Yes | Yes | Yes | Yes | Yes | 8 |
| Greil 2011 | Yes | Yes | Yes | Yes | Yes | Yes | Yes | Yes | 8 |
| Gui 2021 | Yes | Yes | Yes | Yes | Yes | Yes | Yes | Yes | 8 |
| Honarvar 2020 | Yes | Yes | Yes | Yes | Yes | Yes | Yes | Yes | 8 |
| Hui 2017 | Yes | Yes | Yes | Yes | Yes | Yes | Yes | Yes | 8 |
| Ikemoto 2021 | Yes | Yes | Yes | Yes | Yes | Yes | Yes | Yes | 8 |
| Khadim 2019 | Yes | Yes | Yes | Yes | Yes | Yes | Yes | Yes | 8 |
| Khalid2020 | Yes | Yes | Yes | Yes | Yes | Yes | Yes | Yes | 8 |
| Lanskara 2011 | Yes | Yes | Yes | Yes | Yes | Yes | Yes | Yes | 8 |
| Lykeridou 2011 | Yes | Yes | Yes | Yes | Yes | Yes | Yes | Yes | 8 |
| Naab 2013 | Yes | Yes | Yes | Yes | Yes | Yes | Yes | Yes | 8 |
| Namdar 2017 | Yes | Yes | Yes | Yes | No | No | Yes | Yes | 6 |
| Ogawa, 2011 | Yes | Yes | Yes | Yes | No | No | Yes | Yes | 6 |
| Oladeji, 2017 | Yes | Yes | Yes | Yes | No | No | Yes | Yes | 6 |
| Omoaregba 2011(Omoaregba et al. 2011) | No | No | UC | Yes | No | No | Yes | UC | 2 |
| Qadir, 2015 | Yes | Yes | Yes | Yes | Yes | Yes | Yes | Yes | 8 |
| Razzaque 2021 | No | No | Yes | Yes | No | No | Yes | Yes | 4 |
| Rufai, 2022 | Yes | Yes | Yes | Yes | Yes | Yes | Yes | Yes | 8 |
| Shafierizi, 2022 | Yes | Yes | Yes | Yes | No | No | Yes | Yes | 6 |
| Shin, 2021 | Yes | No | Yes | Yes | No | No | Yes | Yes | 5 |
| Sulyman, 2019 | Yes | No | Yes | Yes | No | No | Yes | Yes | 5 |
| Takaki, 2014 | No | No | Yes | Yes | Yes | No | Yes | Yes | 5 |
| Teklemicheal, 2022 | Yes | Yes | Yes | Yes | No | No | Yes | Yes | 6 |
| Tola, 2020 | Yes | Yes | Yes | Yes | No | No | Yes | Yes | 6 |
| Vo, 2019 | Yes | Yes | Yes | Yes | No | No | Yes | Yes | 6 |
| Wang, 2018 | Yes | No | Yes | Yes | No | No | Yes | Yes | 5 |
